# Long noncoding RNA NEAT1 suppresses hepatocyte proliferation in fulminant hepatic failure through increased recruitment of EZH2 to the LATS2 promoter region and promotion of H3K27me3 methylation

**DOI:** 10.1038/s12276-020-0387-z

**Published:** 2020-03-10

**Authors:** Qiang Wang, Lian Liu, Sheng Zhang, Yingzi Ming, Shu Liu, Ke Cheng, Yujun Zhao

**Affiliations:** Transplantation Center, the Third Xiangya Hospital of Central South University, 410013 Changsha, P. R. China

**Keywords:** Biotechnology, Cell biology

## Abstract

Fulminant hepatic failure (FHF) refers to the rapid development of severe acute liver injury with impaired synthetic function and encephalopathy in people with normal liver or well-compensated liver disease. This study aimed to investigate the function of long noncoding RNA (lncRNA) nuclear-enriched abundant transcript 1 (NEAT1) on the proliferation and apoptosis of hepatocytes in FHF. Our results revealed that lncRNA NEAT1 was upregulated in cell and animal models of FHF induced by D-galactosamine (D-GalN)/lipopolysaccharide (LPS). Overexpression of lncRNA NEAT1 resulted in elevated hepatocyte apoptosis and impaired large tumor-suppressor kinase 2 (LATS2) expression and proliferation. Functional analysis revealed that knockdown of lncRNA NEAT1 inhibited hepatocyte apoptosis and induced proliferation both in vitro and in vivo. RNA immunoprecipitation and chromatin immunoprecipitation assays demonstrated that lncRNA NEAT1 recruited enhancer of zeste homolog 2 (EZH2) to the LATS2 promoter and repressed LATS2 expression. Furthermore, ectopic expression of LATS2 increased proliferation and inhibited hepatocyte apoptosis by regulating the Hippo/Yes-associated protein (YAP) signaling pathway. Taken together, our findings indicate that lncRNA NEAT1 might serve as a novel target for FHF therapy due to its regulation of H3K27me3 methylation-dependent promotion of LATS2.

## Liver failure: A NEAT possibility for treatment

A long noncoding RNA molecule, one that does not encode the synthesis of protein, is implicated in acute liver failure (AHF) and might offer a new target for drugs to treat the condition. AHF can be induced by various factors, including viruses, drugs, alcohol abuse, and inherited traits. Ke Cheng, Yujun Zhao and colleagues at Central South University in Changsha, China investigated the role of this RNA, called NEAT1, in cell and animal models of AHF. They identified increased production of NEAT1, which suppressed liver cell proliferation and promoted liver cell death. They also uncovered molecular details of the mechanisms underlying these effects, in which the RNA altered the production and regulatory modification of certain proteins. Further research should investigate the therapeutic possibilities of interfering with NEAT1 activity.

## Introduction

Fulminant hepatic failure (FHF), also known as acute liver failure, is a severe liver injury induced by a variety of stimuli, ranging from viruses, drugs, and alcohol to hereditary metabolic disorders^1^. FHF, accompanied by coagulopathy, hepatic encephalopathy, jaundice, and hydroperitoneum pathologies, is associated with high mortality rates due to the lack of effective therapies except for liver transplantation^2^. However, owing to the constant shortage of liver donors, complications, and high costs, liver transplantation is not a viable option for many patients^3^. It is therefore imperative to identify and develop novel therapeutic approaches to raise the quality of life of patients suffering from FHF. The use of D-galactosamine (D-GalN)/lipopolysaccharide (LPS)-stimulated acute liver injury in mice has been extensively employed as an animal model to investigate the underlying mechanism of FHF and achieve effective target selection^4^. Moreover, apoptosis of hepatocytes is not only a central process that facilitates liver failure but also serves as a trigger for the activation of inflammatory leukocytes, which further exacerbates liver injury^5^; thus regulation of hepatocyte apoptosis might serve as a prospective therapeutic method for FHF.

Long noncoding RNAs (lncRNAs), which are >200 nucleotides, have drawn the attention of numerous researchers due to their involvement in epigenetic control, cell cycle regulation, translation, differentiation, and other important cellular processes^6^. The lncRNA nuclear-enriched abundant transcript 1 (NEAT1) was detected in primary hepatocytes isolated from fibrotic livers and identified as a regulator in the progression of liver fibrosis^7^. Moreover, previous studies have highlighted the role of lncRNA NEAT1 as an oncogene in bladder cancer, colorectal cancer, and hepatocellular carcinoma^8–10^. In addition, lncRNA NEAT1 can bind to enhancer of zeste homolog 2 (EZH2) and regulate the trimethylation of H3K27 in gene promoters in glioblastoma^11^. Notably, co-transcriptional methylation and acetylation under dynamic conditions have been considered for the development of novel treatment modalities^12^. Furthermore, it has also been demonstrated that EZH2 can repress the expression of large tumor-suppressor kinase 2 (LATS2) in non-small-cell lung cancer cells, and we hypothesize a similar action in FHF^13^. LATS2 has further emerged as a key regulator of cell progression due to its broad variety of biological activities in cell cycle regulation and motility and the varied pathological outcomes resulting from its downregulation^14^. Furthermore, YAP and TAZ are major downstream effectors of the Hippo axis and participate in modulating the expression of genes that are vital for cell proliferation and survival^15^. Importantly, YAP/TAZ has been elucidated to be essential for proper liver regeneration, and the Hippo pathway is required for liver regeneration^16^. Deregulation of the Hippo pathway, particularly abnormal YAP hyperactivation, has also been broadly documented in liver physiology and pathology^17^. The current study aims to elucidate the role and mechanism of action of lncRNA NEAT1 in apoptosis in D-GalN/LPS-induced FHF. To explore this, we established cell and animal models of FHF by the administration of D-GalN/LPS and then evaluated various parameters, such as liver damage, hepatocyte apoptosis, and proliferation.

## Material and methods

### Ethical statement

The current study was approved by the Ethics Committee of the Third Xiangya Hospital of Central South University. All animal experiments were strictly designed and carried out in accordance with the Guide for the Care and Use of Laboratory Animals by International Committees. Great efforts were made to minimize the number and suffering of the included animals.

### Cell culture and treatments

The HL-7702 cell line was purchased from Shanghai Cell Bank, Chinese Academy of Sciences (Shanghai, China) and cultured in Dulbecco’s modified Eagle’s medium at 37 °C with 5% CO_2_ in air. In vitro FHF cell models were established by treating HL-7702 cells with D-GalN/LPS (D-GalN: 1 mmol/L; LPS: 10 ng/mL). Previous studies have highlighted that lentiviruses can be applied to knock down nuclear lncRNAs^18–20^. The overexpression lentivirus vector (LV5-GFP) and silencing lentivirus vector (pSIH1-H1-copGFP) were both constructed by Gene Pharma (Shanghai, China). Virus infection was performed 24 h after D-GalN/LPS treatment. Briefly, hepatocytes at the logarithmic growth phase were inoculated in 6-well cell culture plates at a density of 2 × 10^5^ cells/well. When confluence reached 30%, the cells were treated with the corresponding lentivirus. After 48 h of infection, the stably transfected cells were selected by adding 1 μg/mL puromycin to each well. The specific groups included the control, D-GalN/LPS, oe-negative control (NC), oe-NEAT1, oe-LATS2, oe-EZH2, sh-NC, sh-NEAT1, sh-LATS2, sh-EZH2, D-GalN/LPS+oe-NC, D-GalN/LPS+oe-NEAT1, D-GalN/LPS+oe-LATS2, D-GalN/LPS+oe-EZH2, D-GalN/LPS+oe-LATS2, D-GalN/LPS+sh-NC, D-GalN/LPS+sh-NEAT1, and D-GalN/LPS+sh-EZH2 groups. The cells were cultured for another 24–48 h for subsequent experimentation.

### RNA isolation and quantification

Total RNA was extracted using TRIzol reagent (Invitrogen Inc., Carlsbad, CA, USA). Subsequently, agarose gel electrophoresis was performed to detect the integrity of RNA. According to the manufacturer’s instructions, cDNA was synthesized using a PrimeScript^TM^ reverse transcription (RT) reagent kit (RRO37A, TaKaRa, Dalian, Liaoning, China). RT quantitative polymerase chain reaction (RT-qPCR) was performed using an ABI7500 qPCR instrument (ABI Company, Oyster Bay, NY, USA). The primers used are listed in Table 1, and the relative quantification value for each target gene was calculated using a relative quantification method (2^−ΔΔCt^ method), with β-actin serving as the internal control^21^.

**Table 1:** Primer sequences for RT-qPCR.

Gene	Primer sequence
NEAT1 (human)	F: 5′-TTTGTGCTTGGAACCTTGCT-3′
	R: 5′-TCAACGCCCCAAGTTATTTC-3′
LATS2 (human)	F: 5′-ACTTTTCCTGCCACGACTTATTC-3′
	R: 5′-GATGGCTGTTTTAACCCCTCA-3′
NEAT1 (rat)	F: 5′-ACTGCTTGACACCCCATGCC-3′
	R: 5′-CGGTGATGACCACGGCTACC-3′
LATS2 (rat)	F: 5′-CCAACAGCAAGCACCCAGAG-3′
	R: 5′-CGACCGCACCTCCTAACTC-3′
β-Actin (human)	F: 5′-CCAAGGCCAACCGCGAGAAGATGAC-3′
	R: 5′-AGGGTACATGGTGGTGCCGCCAGAC-3′
β-Actin (rat)	F: 5′-AGAGCTACGAGCTGCCTGAC-3′
	R: 5′-AGTACTTGCGCTCAGGAGGA-3′

*RT-qPCR* reverse transcription quantitative polymerase chain reaction, *NEAT1* nuclear-enriched abundant transcript 1, *LATS2* large tumor-suppressor kinase 2, *F* forward, *R* reverse.

### Western blot analysis and coimmunoprecipitation (coIP)

Cytoplasmic and nuclear proteins were isolated using NE-PER cytoplasmic and nuclear extraction reagents (Thermo Fisher Scientific Inc., Waltham, MA, USA) according to the manufacturer’s instructions. The protein concentration was determined using bicinchoninic acid kits (20201 ES76, Yeasen Biotechnology Co. Ltd., Shanghai, China). Equal amounts of protein were then separated by 10% sodium dodecyl sulfate (SDS)–polyacrylamide gel electrophoresis and transferred onto a nitrocellulose membrane (ZY-160FP, ZYSW, Shanghai, China). After being blocked with 5% skim milk at room temperature for 2 h, the membrane was incubated with anti-LATS2 (ab135794, dilution ratio of 1:500), anti-EZH2 (ab191080, dilution ratio of 1:500), anti-trimethylation (me3) of lysine 27 (K27) on histone 3 (H3) (H3K27me3) (ab192985, dilution ratio of 1:1000), anti-YAP1 (ab39361, dilution ratio of 1:1000), and anti-β-actin (ab8227, dilution ratio of 1:1000) overnight at 4 °C. The membranes were then incubated with the secondary antibody horseradish peroxidase (HRP)-conjugated goat anti-rabbit antibody to immunoglobulin G (IgG) (ab20272, dilution ratio of 1:5000) at 37 °C for 1 h. All the aforementioned antibodies were purchased from Abcam Inc. (Cambridge, UK). The protein bands were developed with a chemiluminescence reagent (ECL808–25, Biomiga, San Diego, CA, USA), and images were captured with an X-ray machine (36209ES01, Qcbio Science & Technologies Co., Ltd., Shanghai, China). The relative protein levels were expressed by the image intensity value of the target protein band over the intensity value of the β-actin protein band.

For coIP, 50–75 μg of protein lysates were incubated with anti-YAP1 (ab52771, dilution ratio of 1:50, Abcam) overnight at 4 °C, followed by incubation with 30 μL of protein G Plus-Sepharose (Amersham Pharmacia Biotech, Chicago, IL, USA) at 25 °C for 2–4 h. The beads were pelleted, washed three times in IP buffer, and separated by SDS-polyacrylamide gel electrophoresis. The immune complexes were analyzed by western blot analysis with anti-P73 antibody (ab40658; 1:1000, Abcam).

### RNA immunoprecipitation (RIP)

The experiment was conducted according to the instructions of the Magna RIP RNA-binding protein immunoprecipitation kit (Millipore Corp, Billerica, MA, USA). Briefly, HL-7702 cells were collected by cell scraping and lysed with 100 μL of lysis buffer containing protease inhibitor and ribonuclease inhibitor, and then the protein lysates were incubated with the anti-EZH2 antibody (ab186006, Abcam) for 30 min at 4 °C, followed by the addition of 10–50 μL of protein A/G-beads overnight at 4 °C. After incubation, the protein A/G-bead precipitate was washed 3–4 times with 1 mL of lysis buffer, and RNA was isolated and purified from the precipitate using an RNA extraction method. The interaction between EZH2 and NEAT1 was verified by qPCR assays with NEAT1-specific primers. The following antibodies used in RIP assays were purchased from Abcam: rabbit antibody to EZH2 (ab186006, dilution ratio of 1:500) and rabbit anti-human antibody to IgG (ab109489, dilution ratio of 1:100) as NC.

### Chromatin immunoprecipitation (ChIP) assay

An EZ-Magna ChIP kit (Millipore Corp) was used to conduct the ChIP assay. Briefly, HL-7702 cells were fixed with 4% paraformaldehyde and incubated with glycine for 10 min to generate DNA–protein cross-links. The cells were then lysed with a cell lysis buffer and nuclear lysis buffer and sonicated to produce chromatin fragments of 200–300 bp. Then the lysates were immunoprecipitated with magnetic protein A beads conjugated with the appropriate antibodies. Anti-histone H3 antibody (trimethyl K27; ab6002) and IgG (ab171870) were used as positive and negative controls, respectively. All the aforementioned antibodies were purchased from Abcam except anti-trimethyl-histone H3 (Abnova, Taiwan, China). Finally, the obtained ChIP DNA was analyzed using RT-qPCR.

### 5-Ethynyl-2′-deoxyuridine (EdU) assay

Cells were inoculated in a 24-well plate, incubated with 200 μL of EdU medium (5 μM) for 2 h, and fixed with 50 μL phosphate buffer saline (PBS) containing 4% paraformaldehyde for 30 min at room temperature. Subsequently, the cells were treated with 50 μL of glycine (2 mg/mL) for 5 min, 200 μL of osmotic agent (PBS containing 0.5% Triton X-100) for 10 min, and 200 μL of IX Apollo staining solution at room temperature in the dark for 30 min and then rinsed 2–3 times with 200 μL of osmotic agent (PBS containing 0.5% Triton X-100; 10 min each time). The cells were then counterstained with 4′,6-diamidino-2-phenylindole (DAPI) for 5 min for nuclear staining and observed under a fluorescence microscope.

### Establishment of a rat model of FHF

A total of 105 healthy male Sprague-Dawley rats (specific pathogen- free; aged 7–8 weeks; weighing 190–220 g) were purchased from Better Biotechnology Co., Ltd. (Nanjing, Jiangsu, China). Fifteen rats were used as normal controls, and 15 rats were injected with PBS solution as vehicle controls. The overexpression lentivirus vector (LV5-GFP) and silencing lentivirus vector (pSIH1-H1-copGFP) were both constructed by Gene Pharma. The lentivirus was packaged in 293T cells and cultured in RPMI-1640 medium containing 10% fetal bovine serum. To knock down lncRNA NEAT1 or overexpress LATS2, the remaining 75 rats were not injected (*n* = 15) or subjected to intraperitoneal injections of lentivirus vectors at a concentration of 5 × 10^8^ pfu/100 μL (oe-NC, oe-LATS2, sh-NC, and sh-NEAT1, *n* = 15 each). After 48 h, according to the protocol described in a previous study^22^, the aforementioned 75 rats were intraperitoneally injected with 700 mg/kg GalN (Sigma-Aldrich Chemical Company, St Louis, MO, USA) and 4.5 μg/kg LPS (Sigma-Aldrich Chemical Company) to induce FHF. Subsequently, 6 rats were euthanized from each group at 0 and 12 h after injection to obtain liver tissues.

### Enzyme-linked immunosorbent assay (ELISA) for serum parameter analysis

The supernatants of primary hepatocytes and HL-7702 cells or rat serum were collected to analyze liver function-related parameters. The aspartate aminotransferase (AST), alanine aminotransferase (ALT), and lactate dehydrogenase (LDH) levels in plasma were measured using commercial kits (Sigma-Aldrich).

### Terminal deoxynucleotidyl transferase (TdT)-mediated 2′-deoxyuridine 5′-triphosphate (dUTP) biotin nick end labeling (TUNEL) staining assay

Cell apoptosis in the liver tissues of rats was determined using DeadEnd^TM^ fluorescent-labeling TUNEL assay kits (Promega Corporation, Madison, WI, USA). Liver tissue sections were treated with 2% H_2_O_2_ at room temperature for 10 min and with 20 μg/mL DNase-free proteinase K for 30 min at 37 °C to remove nuclease. The sections were then incubated with 100 μL of equilibrium buffer for 10 min at room temperature, 100 μL of TdT enzyme at 37 °C for 60 min away from light, and finally with the reaction termination solution at room temperature for 10 min. The cells were counterstained with DAPI solution (10 mg/mL) at room temperature for 5 min prior to analysis by laser confocal microscopy (Nikon, Japan). Five high-power visual fields (×400) were randomly selected from each group. The images were then analyzed by the ImageProPlus image analysis and processing system (Motic Med 6.0; Motic Image Technology Co., Ltd., Beijing, China). The apoptotic rate is represented as follows: (number of green stained cells/number of blue stained cells) × 100%.

The apoptosis of hepatocytes from rat liver was measured using TUNEL staining. The samples fixed with formaldehyde were dehydrated, paraffin embedded, and sectioned. Next, the sections were dewaxed 2 times with xylene (5 min each time) and hydrated with gradient ethanol. According to the instructions of the TUNEL test kit (Roche, Basel, Switzerland), 50 μL of TUNEL reaction solution (the ratio of enzyme concentration solution to label solution was 1:9) was added and incubated for 50 min. After incubation with 50 μL of converting agent peroxidase at 37 °C for 30 min, the sections were incubated with 100 μL of diaminobenzidine (DAB) working solution for 10 min, counterstained with hematoxylin for 3 s, and then sealed with neutral gum. The staining was observed under a microscope. Five visual fields were selected from each group. The apoptotic rate in each visual field was calculated as follows: (the number of apoptotic cells/the number of total cells) × 100%.

### Hematoxylin–eosin (HE) staining

After formaldehyde fixation, rat liver tissues were dehydrated with gradient alcohol (70%, 80%, 90%, 95%, and 100%, 5 min each time) and cleared 2 times with xylene 10 min each time. Following wax immersion and paraffin embedding, the tissues were sliced into 4-μm-thick sections and placed on glass slides. After baking at 60 °C for 1 h, the sections were stained sequentially with HE solution for 3 min. Next, the sections were dehydrated, cleared, and sealed with neutral gum. Finally, the sections were observed under an optical microscope to visualize the results of the HE staining (×40; Olympus Optical Co., Ltd., Tokyo, Japan).

### Immunohistochemistry assay

Paraffin-embedded sections of liver tissues were dewaxed and hydrated with gradient ethanol. Antigen retrieval was performed with the use of a microwave, and endogenous peroxidase activity was neutralized with 3% hydrogen peroxide. Subsequently, the sections were incubated with a rabbit polyclonal antibody to LATS2 (ab135794, dilution ratio of 1:50) and rabbit antibody to Ki67 (ab15580, dilution ratio of 1:200) overnight at 4 °C. The HRP-conjugated goat anti-rabbit antibody to IgG (ab6721, dilution ratio of 1:10000) was used as the secondary antibody. The sections were developed with DAB, counterstained with hematoxylin, and sealed. Five visual fields (×400) were randomly selected from each group to count the number of positive cells. The positive cell rate = (the number of positive cells/the total number of cells) × 100%.

### Statistical analysis

All statistical analyses were performed using the SPSS 21.0 software (IBM Corp, Armonk, NY, USA). Measurement data with a normal distribution are expressed as the mean ± standard deviation. The significance of the differences between groups was tested with independent sample *t* test. Multigroup comparisons were conducted using one-way analysis of variance, followed by Tukey’s post hoc test. A value of *p* < 0.05 was indicative of statistical significance.

## Results

### LncRNA NEAT1 was upregulated and LATS2 was downregulated in FHF

**Fig. 1:**
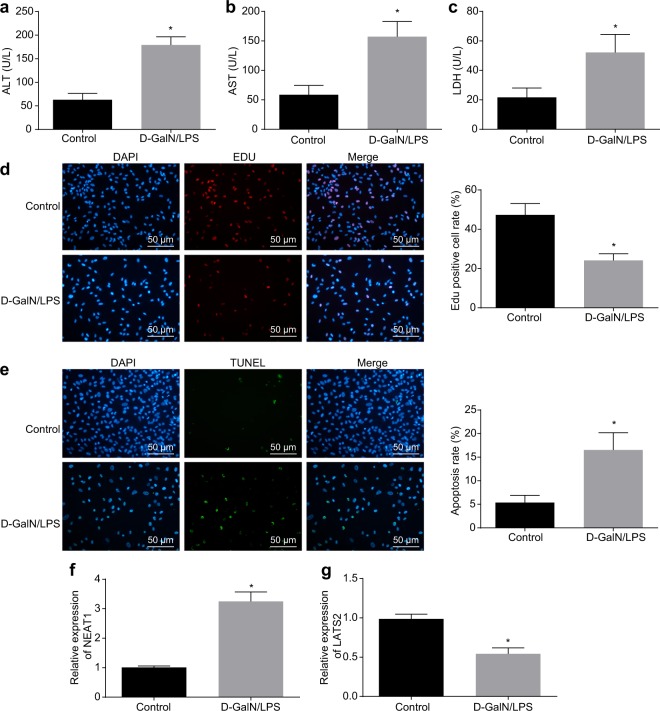
Apoptosis and lncRNA NEAT1 expression increased in D-GalN/LPS-treated cells, whereas LATS2 expression decreased. ALT, AST, and LDH expression patterns in HL-7702 cells with or without D-GalN/LPS treatment (**a**–**c**). The proliferation of HL-7702 cells was determined by EdU assay (×200) (**d**). The apoptosis of HL-7702 cells was determined by TUNEL staining (×200) (**e**). The lncRNA NEAT1 expression patterns in HL-7702 cells determined by RT-qPCR (**f**). The mRNA expression patterns of LATS2 in HL-7702 cells determined by RT-qPCR (**g**). Control, HL-7702 cells without treatment; D-GalN/LPS, HL-7702 cells treated with D-GalN/LPS; *p < 0.05 vs. control. Statistical data are described as mean ± standard deviation. Independent sample *t* test was used for comparisons between two groups. The experiment was repeated three times independently. RT-qPCR reverse transcription quantitative polymerase chain reaction, lncRNA NEAT1 long noncoding RNA nuclear-enriched abundant transcript 1, LATS2 large tumor-suppressor kinase 2, D-GalN/LPS D-galactosamine/lipopolysaccharide, AST aspartate aminotransferase, ALT alanine aminotransferase, LDH lactate dehydrogenase, ELISA enzyme-linked immunosorbent assay, EdU 5-ethynyl-2′-deoxyuridine, TUNEL terminal deoxynucleotidyl transferase (TdT)-mediated 2′-deoxyuridine 5′-triphosphate (dUTP) biotin nick end labeling.

A previous report showed that the expression of lncRNA NEAT1 was significantly increased in non-alcoholic fatty liver disease (NAFLD) models in vivo and in vitro, while downregulation of lncRNA NEAT1 alleviated NAFLD through the mammalian target of rapamycin/S6K1 signaling pathway^23^. In addition, it is also known that lncRNA NEAT1 accelerates the progression of hepatic fibrosis by regulating microRNA-122 and Kruppel-like factor 6^7^. To investigate the expression patterns of lncRNA NEAT1 and LATS2 in FHF cell models, we used D-GalN/LPS to treat HL-7702 cells to establish FHF cell models. ELISA was applied to test liver function-related parameters (ALT, AST, and LDH) in culture medium 24 h after treatment. The expression of ALT, AST, and LDH in D-GalN/LPS-treated cells was significantly upregulated (Fig. 1a–c), indicating the successful establishment of the FHF cell model. Next, EdU and TUNEL assays were used to assess the proliferation and apoptosis of HL-7702 cells, respectively. Cells treated with D-GalN/LPS demonstrated significantly inhibited proliferation and significantly increased apoptosis rates compared to untreated cells (Fig. 1d, e). Moreover, the expression patterns of lncRNA NEAT1 and LATS2 were determined, which revealed that the expression levels of lncRNA NEAT1 were elevated significantly, whereas those of LATS2 were decreased significantly after D-GalN/LPS treatment (Fig. 1f, g). These results suggested that D-GalN/LPS treatment could cause hepatocyte injury in FHF and induce the expression of lncRNA NEAT1 while blocking the expression of LATS2.

### Knockdown of lncRNA NEAT1 inhibited apoptosis and induced cell proliferation by modulating LATS2

**Fig. 2:**
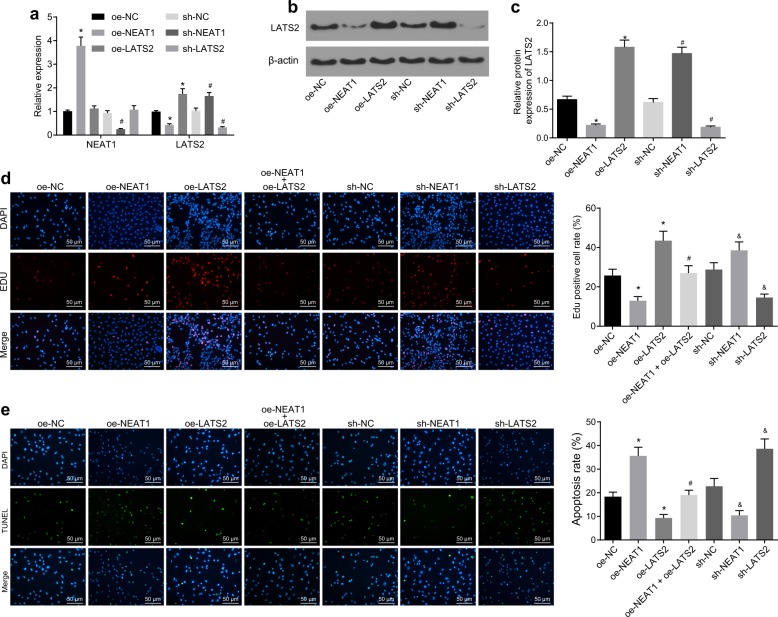
Knockdown of lncRNA NEAT1 inhibits apoptosis and induces cell proliferation by modulating LATS2. The expression patterns of lncRNA NEAT1 and LATS2 in cells treated with oe-NEAT1, oe-LATS2, sh-NEAT1, or sh-LATS2 evaluated by RT-qPCR (**a**). The protein expression patterns of LATS2 in cells treated with oe-NEAT1, oe-LATS2, sh-NEAT1, or sh-LATS2 evaluated by western blot analysis (**b**). Quantification of protein expression from **b** (**c**). *p < 0.05 vs. the oe-NC group; ^#^*p* < 0.05 vs. the sh-NC group. The proliferation of HL-7702 cells treated with sh-NEAT1, sh-LATS2, oe-NEAT1, and/or oe-LATS2 measured by the EdU assay (×200) (**d**). The apoptosis of HL-7702 cells treated with sh-NEAT1, sh-LATS2, oe-NEAT1, and/or oe-LATS2 measured by TUNEL staining (×200) (**e**). *p < 0.05 vs. the oe^-^NC group; ^#^*p* < 0.05 vs. the oe-LATS2 group; ^&^*p* < 0.05 vs. the sh-NC group. oe-NC group, HL-7702 cells treated with oe-NC; oe-NEAT1 group, HL-7702 cells treated with oe-NEAT1; oe-LATS2 group, HL-7702 cells treated with oe-LATS2; sh-NEAT1 group, HL-7702 cells treated with sh-NEAT1; sh-LATS2 group, HL-7702 cells treated with sh-LATS2; oe-NEAT1+oe-LATS2 group, HL-7702 cells treated with both oe-NEAT1 and oe-LATS2. Statistical data are described as mean ± standard deviation. One-way analysis of variance was used for comparisons among multiple groups, followed by Tukey’s post hoc test. The experiment was repeated three times independently. RT-qPCR reverse transcription quantitative polymerase chain reaction, lncRNA NEAT1 long noncoding RNA nuclear-enriched abundant transcript 1, LATS2 large tumor-suppressor kinase 2, EdU 5-ethynyl-2′-deoxyuridine, TUNEL terminal deoxynucleotidyl transferase (TdT)-mediated 2′-deoxyuridine 5′-triphosphate (dUTP) biotin nick end labeling, NC negative control.

Subsequently, we investigated the effect of lncRNA NEAT1 and LATS2 on apoptosis and proliferation in the D-GalN/LPS-induced FHF cell model. First, we overexpressed or blocked lncRNA NEAT1/LATS2 by lentivirus infection. RT-qPCR and western blot analysis were subsequently performed to verify the infection efficiency (Fig. 2a–c). Overexpression of lncRNA NEAT1 significantly inhibited the mRNA and protein expression of LATS2, while silencing of lncRNA NEAT1 produced the opposite results. However, overexpression or silencing of LATS2 exerted negligible effects on the expression of lncRNA NEAT1. These results suggested that lncRNA NEAT1 could regulate the expression of LATS2 in FHF cells. Thereafter, EdU and TUNEL assays were conducted to examine the proliferation and apoptosis of HL-7702 cells treated with D-GalN/LPS. The results revealed that the proliferation of HL-7702 cells treated with oe-NEAT1 was significantly lower, while apoptosis was higher relative to that in the oe-NC group, while the opposite trends were observed in cells treated with sh-NEAT1. In contrast, overexpression of LATS2 promoted cell proliferation and inhibited cell apoptosis (Fig. 2d, e). Furthermore, when compared to oe-NEAT1 treatment alone, combined treatment with oe-NEAT1 and oe-LATS2 significantly restored cell proliferation and decreased apoptosis (Fig. 2d, e). The aforementioned results indicated that knockdown of lncRNA NEAT1 expression and overexpression of LATS2 could suppress cell apoptosis and induce cell proliferation.

### Downregulation of lncRNA NEAT1 increased LATS2 expression in FHF rats

**Fig. 3:**
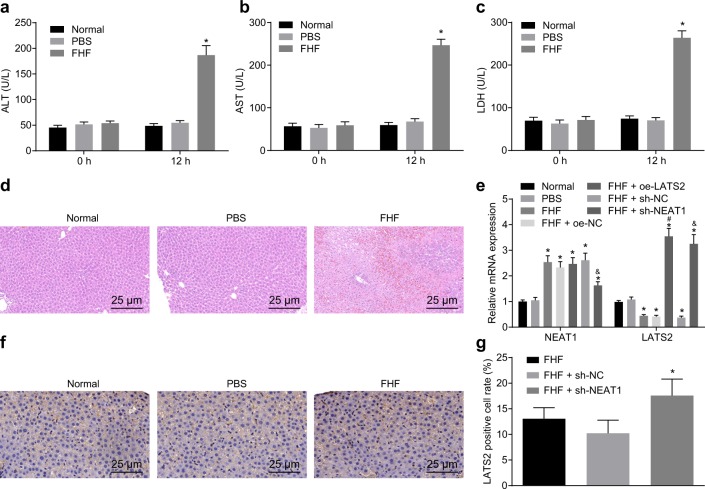
Downregulation of lncRNA NEAT1 restores LATS2 expression in FHF rats. ALT, AST, and LDH expression patterns in the FHF rat model measured by ELISA (**a**–**c**). Liver pathology in FHF rats tested by HE staining (**d**). The lncRNA NEAT1 and LATS2 expression patterns in liver tissues of FHF rats evaluated by RT-qPCR (**e**). Immunohistochemical staining of LATS2 expression in liver tissues of FHF rats (×400) (**f**). Quantification of the results of immunohistochemical staining (**g**). *p < 0.05 vs. the normal group; ^#^*p* < 0.05 vs. the FHF+oe-NC group; ^&^*p* < 0.05 vs. the FHF+sh-NC group. Normal group, normal rats without injection; PBS group, normal rats injected with PBS; FHF group, FHF rats induced by D-GalN/LPS injection; FHF+oe-NC group, FHF rats treated with oe-NC; FHF+oe-LATS2 group, FHF rats treated with oe-LATS2; FHF+sh-NC group, FHF rats treated with sh-NC; FHF+sh-NEAT1 group, FHF rats treated with sh-NEAT1. Statistical data are described as mean ± standard deviation. One-way analysis of variance was used for comparisons among multiple groups, followed by Tukey’s post hoc test. *n* = 6. RT-qPCR reverse transcription quantitative polymerase chain reaction, lncRNA NEAT1 long noncoding RNA nuclear-enriched abundant transcript 1, LATS2 large tumor-suppressor kinase 2, D-GalN/LPS D-galactosamine/lipopolysaccharide, AST aspartate aminotransferase, ALT alanine aminotransferase, LDH lactate dehydrogenase, ELISA enzyme-linked immunosorbent assay, NC negative control, FHF fulminant hepatic failure.

To study the expression patterns and role of lncRNA NEAT1 in FHF in vivo, we established a male FHF rat model by tail vein injections of D-GalN/LPS. No abnormal reactions were observed in rats after normal saline administration. After D-GalN/LPS administration, the general condition of rats deteriorated gradually with the administration time (for example, the response to external stimuli was slow, animals were sluggish and even in a state of coma). The expression of ALT, AST, and LDH in FHF rats was significantly higher than that in normal rats with or without PBS injection (Fig. 3a–c). The results of HE staining demonstrated that the liver tissues of normal rats with or without PBS injection were normal with clear hepatic lobule structures and regular hepatic sinusoidal structure. In FHF rats, apparent liver injury, characterized by cell edema, hepatocyte lysis, diffuse necrosis, and inflammatory cell infiltration, was observed (Fig. 3d), suggesting that the FHF rat model was successfully established. Subsequently, we determined the expression patterns of lncRNA NEAT1 and LATS2 in the liver tissue of rats treated with D-GalN/LPS for 12 h. It was found that FHF rats presented with significantly higher expression of lncRNA NEAT1 compared to normal rats with or without PBS injection, whereas the expression of LATS2 was decreased (Fig. 3e).

Furthermore, we modulated the expression of lncRNA NEAT1 and LATS2 in rats via upregulation or downregulation by intravenous injection of lentivirus. The expression of LATS2 in the liver tissue of FHF rats injected with oe-LATS2 was significantly higher than that in FHF rats injected with oe-NC, while lncRNA NEAT1 expression was significantly decreased in FHF rats injected with sh-NEAT1 relative to FHF rats injected with sh-NC (Fig. 3e). Subsequently, we examined the expression of LATS2 in the rat liver using immunohistochemistry. In comparison with those in FHF rats with or without sh-NC treatment, LATS2 protein levels in FHF rats administered sh-NEAT1 were significantly higher (Fig. 3f, g). All these results suggested that NEAT1 negatively regulated the expression of LATS2 in vivo.

### Downregulation of lncRNA NEAT1 or overexpression of LATS2 inhibited the progression of FHF in vivo

**Fig. 4:**
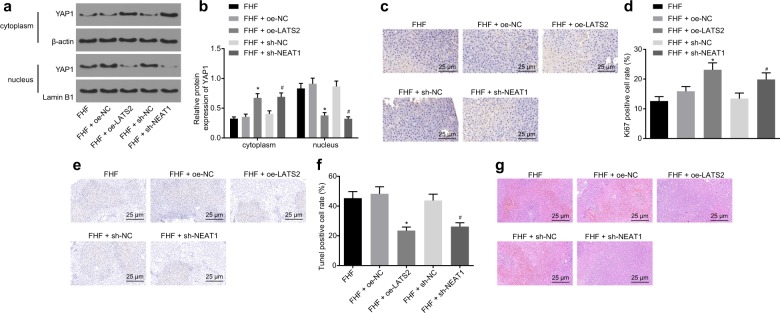
Downregulation of lncRNA NEAT1 or overexpression of LATS2 inhibits the progression of FHF in vivo. The protein expression patterns of YAP1 in the nucleus/cytoplasm of rat liver determined by western blot analysis (**a**, **b**). Immunohistochemical staining of Ki67 expression in the livers of FHF rats (×400) (**c**, **d**). Apoptosis in the livers of FHF rats observed by TUNEL staining (×400) (**e**, **f**). The pathological changes in the livers of FHF rats detected by HE staining (×400) (**g**). *p < 0.05 vs. the FHF+oe-NC group; ^#^*p* < 0.05 vs. the FHF+sh-NC group. FHF group, FHF rats induced by D-GalN/LPS injection; FHF+oe-NC group, FHF rats treated with oe-NC; FHF+oe-LATS2 group, FHF rats treated with oe-LATS2; FHF+sh-NC group, FHF rats treated with sh-NC; FHF+sh-NEAT1 group, FHF rats treated with sh-NEAT1. Statistical data are described as mean ± standard deviation. One-way analysis of variance was used for comparisons among multiple groups, followed by Tukey’s post hoc test. *n* = 6 in each group. D-GalN/LPS D-galactosamine/lipopolysaccharide, lncRNA NEAT1 long noncoding RNA nuclear-enriched abundant transcript 1, LATS2 large tumor-suppressor kinase 2, FHF fulminant hepatic failure, TUNEL terminal deoxynucleotidyl transferase (TdT)-mediated 2′-deoxyuridine 5′-triphosphate (dUTP) biotin nick end labeling, HE hematoxylin–eosin.

We determined that lncRNA NEAT1 was highly expressed and could inhibit the expression of LATS2 in the FHF rat model. Next, we shifted our focus to investigating whether lncRNA NEAT1 and LATS2 also affect FHF progression in vivo and performed a series of experiments. Western blot analysis was used to assess the protein expression patterns of YAP1 in rat liver tissues. The results revealed that downregulation of lncRNA NEAT1 or overexpression of LATS2 inhibited the expression of YAP1 in the nucleus (Fig. 4a, b). Next, the expression patterns of Ki67 in liver tissues of rats in each group were measured by immunohistochemical staining. The expression of Ki67 in FHF rats injected with sh-NEAT1 or oe-LATS2 was significantly higher than that in FHF rats injected with sh-NC or oe-NC (Fig. 4c, d). These findings suggested that downregulation of lncRNA NEAT1 or overexpression of LATS2 could promote the proliferation of hepatocytes in vivo. The TUNEL assay demonstrated that silencing lncRNA NEAT1 or overexpression of LATS2 significantly inhibited apoptosis of hepatocytes in FHF rats (Fig. 4e, f). Moreover, histological examination revealed that decreased lncRNA NEAT1 or elevated LATS2 alleviated the liver injury of FHF rats relative to FHF rats injected with sh-NC or oe-NC, which was characterized by a decreased hepatocyte edema and improved degeneration of hepatic sinusoidal dilatation and infiltration of necrotic and inflammatory cells (Fig. 4g). Altogether, these findings indicated that downregulation of lncRNA NEAT1 or overexpression of LATS2 inhibited the progression of FHF in vivo.

### LncRNA NEAT1 regulated LATS2 expression by recruiting EZH2 to the LATS2 promoter region

**Fig. 5:**
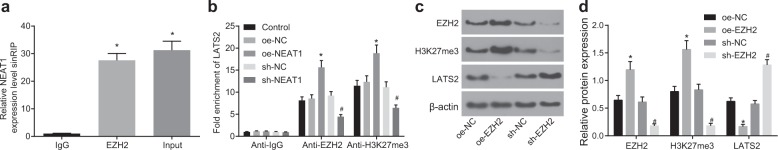
LncRNA NEAT1 recruits EZH2 to the LATS2 promoter and represses LATS2 expression. The interaction between lncRNA NEAT1 and EZH2 was verified by RIP assay. *p < 0.05 vs. the IgG group (**a**). The enrichment of EZH2 and H3K27me3 on the LATS2 promoter region determined by ChIP (**b**). The protein expression patterns of EZH2, H3K27me3, and LATS2 in response to overexpression or knockdown of EZH2 measured by western blot analysis (**c**). Quantification of protein expression in **c** (**d**). Control group, HL-7702 cells without treatment; oe-NC group, HL-7702 cells treated with oe-NC; sh-NC group, HL-7702 cells treated with sh-NC; oe-EZH2 group, HL-7702 cells treated with oe-EZH2; sh-EZH2 group, HL-7702 cells treated with sh-EZH2. Statistical data are described as mean ± standard deviation. One-way analysis of variance was used for comparisons among multiple groups, followed by Tukey’s post hoc test. The experiment was repeated three times independently. lncRNA NEAT1 long noncoding RNA nuclear-enriched abundant transcript 1, LATS2 large tumor-suppressor kinase 2, EZH2 enhancer of zeste homolog 2, NC negative control, RIP RNA immunoprecipitation, ChIP chromatin immunoprecipitation.

Previous studies have reported that lncRNA MEG3 promotes the ubiquitination of EZH2 and inhibits the proliferation and invasion of gallbladder carcinoma cells by regulating LATS2^24^. Moreover, it is also known that lncRNA NEAT1 can inhibit E-cadherin expression by recruiting EZH2 to the E-cadherin promoter^25^. Our results showed that the expression of lncRNA NEAT1 and LATS2 in FHF was negatively correlated and that lncRNA NEAT1 downregulated the expression of LATS2 and ultimately modulated the proliferation and apoptosis of HL-7702 cells. Therefore, we speculated that lncRNA NEAT1 might inhibit the expression of LATS2 by recruiting EZH2 to the LATS2 promoter region in FHF. Online analysis (http://lncrna.smu.edu.cn/show/DNATriplex) revealed that there were potential interaction regions between lncRNA NEAT1 and the LATS2 promoter. Next, we performed RIP assays using an antibody against EZH2. As shown in Fig. 5a, lncRNA NEAT1 levels were evidently higher in EZH2-RNA precipitates than in IgG-RNA precipitates, suggesting that lncRNA NEAT1 could interact with the EZH2 protein. As a histone methyltransferase, EZH2 specifically participates in covalent modification of the histone tail, especially H3K27me3, which is an inhibitory marker found in several gene promoters^26^. Furthermore, we conducted a ChIP assay to determine whether EZH2 regulated the expression of LATS2 by methylation of H3K27. The results revealed that compared with that in cells treated with sh-NC, the enrichment of the LATS2 promoter in cells treated with sh-NEAT1 was significantly decreased after pull-down by an antibody against EZH2 or H3K27me3, while the opposite trends were observed in cells treated with oe-NEAT1 compared to those treated with oe-NC (Fig. 5b). It was therefore suggested that EZH2 or H3K27me3 could bind to the LATS2 promoter and be negatively regulated by LATS2. Subsequently, we determined the effect of EZH2 on the expression of LATS2. The results showed that overexpression of EZH2 significantly promoted the expression of EZH2 and H3K27me3 but significantly inhibited that of LATS2. However, knockdown of the EZH2 gene generated the opposite results (Fig. 5c, d). Taken together, these results indicated that lncRNA NEAT1 regulated LATS2 through EZH2-mediated H3K27 methylation.

### The lncRNA NEAT1/EZH2/LATS2 axis activated the Hippo/YAP signaling pathway

**Fig. 6:**
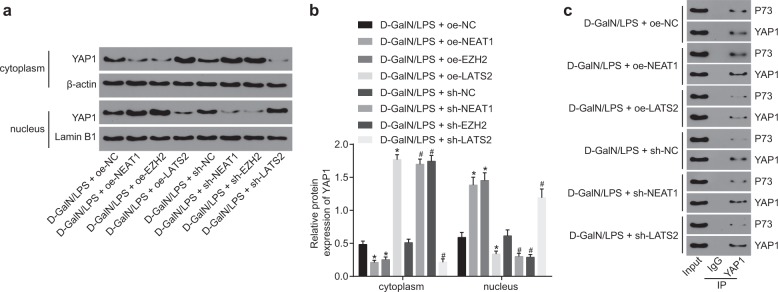
LncRNA NEAT1/EZH2/LATS2 activates the Hippo/YAP signaling pathway. The protein expression patterns of YAP1 in HL-7702 cells treated with D-GalN/LPS quantified by western blot analysis (**a**). Quantification of protein expression in **a** (**b**). The binding relationship between YAP1 and P73, assessed by coIP (**c**). *p < 0.05 vs. the D-GalN/LPS+oe-NC group; ^#^*p* < 0.05 vs. the D-GalN/LPS+sh-NC group. D-GalN/LPS+oe-NC group, HL-7702 cells treated with oe-NC in the presence of D-GalN/LPS treatment; D-GalN/LPS+sh-NC group, HL-7702 cells treated with sh-NC in the presence of D-GalN/LPS treatment; D-GalN/LPS+oe-NEAT1 group, HL-7702 cells treated with oe-NEAT1 in the presence of D-GalN/LPS treatment; D-GalN/LPS+oe-LATS2 group, HL-7702 cells treated with oe-LATS2 in the presence of D-GalN/LPS treatment; D-GalN/LPS+sh-NEAT1 group, HL-7702 cells treated with sh-NEAT1 in the presence of D-GalN/LPS treatment; D-GalN/LPS+sh-LATS2 group, HL-7702 cells treated with sh-LATS2 in the presence of D-GalN/LPS treatment. Statistical data are described as mean ± standard deviation. One-way analysis of variance was used for comparisons among multiple groups, followed by Tukey’s post hoc test. The experiment was repeated three times independently. lncRNA NEAT1 long noncoding RNA nuclear-enriched abundant transcript 1, LATS2 large tumor-suppressor kinase 2, D-GalN/LPS, D-galactosamine/lipopolysaccharide, NC negative control, coIP coimmunoprecipitation.

LATS2 is one of the central kinases in the Hippo signaling pathway and plays a major role in cell proliferation by interacting with the downstream transcription coactivators YAP and TAZ^27^. To study the downstream regulatory mechanisms, we detected the expression patterns of the Hippo/YAP pathway-related protein YAP1 in HL-7702 cells treated with D-GalN/LPS. It was found that, after D-GalN/LPS treatment, silencing of lncRNA NEAT1 or EZH2 and overexpression of LATS2 significantly promoted the expression of YAP1 in the cytoplasm compared to treatment with oe-NC or sh-NC. However, the expression of YAP1 in the nucleus was observed to be significantly decreased. Overexpression of lncRNA NEAT1 or EZH2 and silencing of LATS2 significantly promoted the expression of YAP1 in the nucleus (Fig. 6a, b). CoIP assays revealed that downregulation of lncRNA NEAT1 and upregulation of LATS2 in the presence of D-GalN/LPS treatment significantly inhibited the interaction between YAP1 and P73 relative to the control groups (Fig. 6c). In addition, overexpression of lncRNA NEAT1 and silencing of LATS2 significantly enhanced the binding of YAP1 to P73 (Fig. 6c), indicating that LATS2 may function by mediating the interaction between YAP1 and P73. Thus these findings suggested that lncRNA NEAT1 regulated LATS2 through the Hippo/YAP pathway.

## Discussion

As a fatal complication of acute hepatic injury, FHF occurs unpredictably and is marked by the unexpected onset of hepatic encephalopathy and impaired hepatic function^28^. Interestingly, various lncRNAs, such as H19, HOTAIR, and MALAT-1, and their association with liver diseases have been highlighted as potential markers for disease progression or prognosis^29^. Moreover, D-GalN/LPS-stimulated acute liver injury mouse models have been widely regarded as effective animal models for investigating the underlying mechanisms of FHF and effective target selection^4^. In the current study, we successfully established a D-GalN/LPS-induced FHF rat model and investigated the effects of lncRNA NEAT1 on the apoptosis and proliferation of hepatocytes in vitro and in vivo. Furthermore, the underlying mechanisms were explored. Our findings demonstrated that lncRNA NEAT1 was upregulated both in vivo and in vitro after D-GalN/LPS treatment, which was in accordance with the results from a previous study^30^. In addition, elevated expression of lncRNA NEAT1 has also been documented in NAFLD rat models and free fatty acid-treated rat hepatic cells^23^. Similarly, lncRNA NEAT1 is expressed at significantly high levels in tissues and cell lines of breast cancer, and its knockdown caused a decrease in cell proliferation and EZH2 expression^31^. Interestingly, knockdown of EZH2 also resulted in increased LATS2 expression in gastric cancer cells^32^. A previous study further demonstrated that transduction of LATS2, a component of the Hippo signaling pathway, inhibited the oncoprotein YAP through phosphorylation, thus suppressing malignant mesothelioma cell growth^33^. In addition, silencing of LATS2 in mouse models accelerated tumorigenesis, confirming the existence of the negative control of YAP mediated through LATS2^34^. Another study has also suggested that LATS could regulate cell proliferation, death, and migration independently of the Hippo pathway^35^. Furth et al. reported the involvement of LATS2 in breast cancer cell migration by altering p53 function^36^.

The current study also showed that lncRNA NEAT1 could recruit EZH2 to the LATS2 promoter region and induce the production of H3K27me3, consequently inhibiting the expression of LATS2. In line with our results, the lncRNA PCAT6 has been found to function as an oncogene via EZH2-mediated repression of LATS2 in non-small-cell lung cancer^37^. Adding to its beneficial properties, silencing of EZH2 has also been suggested to be closely correlated with cell proliferation and apoptosis and to promote H3K27me3 and repress gene transcription specifically^38^. Koike et al. demonstrated that the loss of EZH2 is responsible for catalytic enhancement of H3K27me3, leading to significant declines in total liver volume, the number of liver parenchymal cells, and the population of hepatic progenitor cells in a mouse model^39^. In addition, another study further illustrated that deletion of LATS2 results in repressed H3K27me3 expression, while a threshold amount of EZH2 was essential to restore H3K27me3 expression^40^. Interestingly, our findings demonstrated that lncRNA NEAT1 modulated LATS2 expression through increased recruitment of EZH2 to the LATS2 promoter and promoted the methylation of H3K27 to H3K27me3. Likewise, it has also been indicated that lncRNA plasmacytoma variant translocation 1 can recruit EZH2 to elevate the accumulation of H3K27me3^41^, which further validates our results.

Furthermore, we investigated the molecular mechanism behind LATS2 modulation by lncRNA NEAT1. Apoptosis is an important cellular pathological process occurring in D-GalN/LPS-stimulated liver injury; therefore, we explored the mechanism responsible for lncRNA NEAT1 regulation in hepatocyte apoptosis. The Hippo signaling pathway is a well-known mediator of mammalian liver growth and an effective suppressor of liver tumor development^42^. Previously, Cottini et al. explained that genetic inhibition or diminished expression of the Hippo co-transcription factor YAP1 could prevent tumor cells from undergoing the pathologic process of apoptosis^43^. Moreover, YAP1 also possesses the ability to modulate stem cell self-renewal and differentiation and tissue homeostasis, as well as upregulate p73-dependent pro-apoptotic gene transcription^44^. Specifically, the interaction between LATS2 and YAP1 resulted in YAP1 dephosphorylation and nuclear translocation, thereby protecting YAP1 from ubiquitination in the cytoplasm and inducing the expression of pro-proliferation genes in colorectal cancer by working as a transcriptional coactivator^45^.

**Fig. 7:**
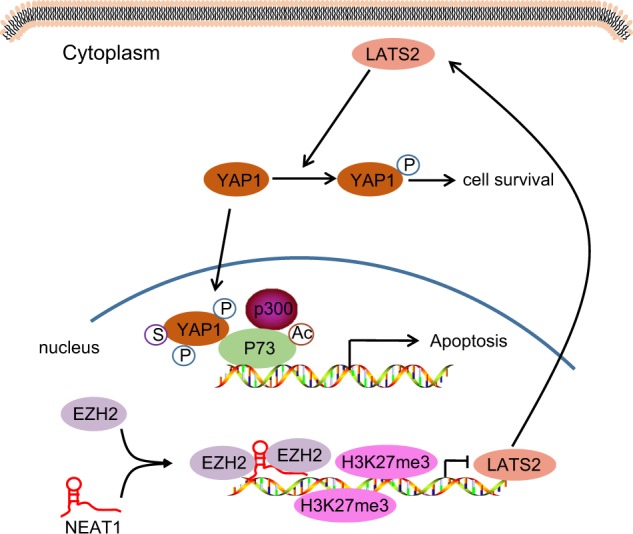
Proposed underlying mechanism of action of lncRNA NEAT1 in the apoptosis and proliferation of hepatocytes in FHF. In FHF hepatocytes, lncRNA NEAT1 recruits EZH2 to the promoter region of LATS2 and induces the production of H3K27me3, thus repressing the expression of LATS2. Upon inhibition of the LATS2 expression, YAP1 could not be activated and enter the nucleus, which results in binding to P73 and induces the apoptosis mediated by P73.

In conclusion, our data revealed that overexpression of lncRNA NEAT1 is associated with downregulation of proliferation and induction of apoptosis and liver damage (Fig. 7). Thus knockdown of lncRNA NEAT1 may serve as a potential therapeutic target for FHF in the future. However, it has been reported that lncRNA NEAT1 can affect glioblastoma cell growth and invasion by inducing trimethylation of H3K27 in the promoter regions of Axis inhibition protein 2, inhibitor of β-catenin and T cell factor, and glycogen synthase kinase 3B or by regulating EZH2 levels^11^. Hence, further investigations are required to explore the effects of lncRNA NEAT1 on these molecules and their methylation in FHF to fully maximize their beneficial effects therapeutically.
